# A narrative review on the therapeutic potential of stem cells in neurodegenerative diseases: advances, insights, and challenges

**DOI:** 10.1097/MS9.0000000000004490

**Published:** 2025-12-09

**Authors:** Tirath Patel, Fathimathul Henna, Iman Sharif, Inaya Javed, Fatima Mustafa, Hannan Sharif, Fatima Nasir, Mirat Javaid, Samman Fatima Usman, Christopher Hanani, Nikhilesh Anand

**Affiliations:** a Department of Neurosurgery, Trinity Medical Sciences University School of Medicine, Saint Vincent and the Grenadines; b Dubai Medical College for Girls, Dubai, UAE; c Akhtar Saeed Medical and Dental College, Lahore, Pakistan; d Texas Institute for Graduate Medical Education and Research, USA; e Department of Neurology, Henry Ford Health, USA; f Department of Medical Education, University of Texas Rio Grande Valley, Edinburg, TX, United States of America

**Keywords:** cell-based therapy, exosomes, neurodegenerative disorders, regenerative medicine, stem cell therapy

## Abstract

**Background:**

Neurodegenerative diseases (NDs) such as Parkinson’s disease (PD), Alzheimer’s disease (AD), amyotrophic lateral sclerosis (ALS), Huntington’s disease (HD) are set apart by progressive neuronal loss and concomitant functional decline. Traditional therapies are equipped with only symptomatic relief, devoid of neurorestorative properties. Stem-cell-based therapies have the potential to revolutionize neurological care by replenishing lost cells, mitigating inflammation, and fostering a neuroprotective environment.

**Objectives:**

This narrative review aims to appraise the treatment potential of various stem cell types in managing NDs, highlighting their functional pathways, delivery methods, and current experimental validation.

**Methods:**

A comprehensive literature search was carried out based on data retrieved from PubMed, The Cochrane Library, and ClinicalTrials.gov. Thirty-one studies that fulfill PICO criteria and only English-language publications are incorporated in this review. No part of the study design, data collection, analysis, or interpretation was conducted using artificial intelligence.

**Results:**

Stem cells, including embryonic stem cells, mesenchymal stem cells (MSCs), induced pluripotent stem cells, and neural stem cells, possess distinctive regenerative properties. MSC-derived exosomes can traverse the blood–brain barrier and improve nerve cell longevity. Administration routes such as intravenous, intranasal, and direct brain transplantation are being studied. Neurodegenerative conditions such as PD, AD, HD, and ALS have been widely studied for therapeutic benefits.

**Conclusion:**

Regardless of their potential, stem cell therapies raise health risks, including neoplastic growth and immunological incompatibility, alongside bioethical issues. Developments in genetic modification, nanotechnology, and preconditioning strategies are being analyzed to optimize outcomes. Long-term research, harmonization of protocols, and extended patient follow-up are essential for the safe and effective development of medical applications.

## Introduction

Neurodegenerative diseases (NDs) arise from progressive neuron loss. Major examples include Parkinson’s disease (PD), amyotrophic lateral sclerosis (ALS), and Alzheimer’s disease (AD)^[1]^. Although the affected areas of the brain may differ from disease to disease, the core pathology of neuronal degeneration is the inflammation of neurons caused by oxidative stress. Proinflammatory phagocytic microglia, reactive astrocytosis, dystrophic neurites, and tau-positive threads contribute to inflammation, leading to neuronal loss in vulnerable brain areas^[2,3]^. The activation of microglia and astroglia serves as the primary cellular regulatory mechanism in NDs^[4]^.

Despite decades of studies, it is still challenging to create an effective treatment plan for NDs^[1,5]^. Several factors contribute, including the difficulty in precise drug delivery methods to affected brain tissue, targeting specific inflammatory microglial and astrocytic phenotypes, and challenges in modulating key messenger proteins to restore neuronal signaling pathways^[1]^. The current approach includes medication, surgery, and rehabilitation. These therapies can only provide temporary symptomatic relief, with little to no chance of restoring impaired neuronal functionality^[6,7]^. However, a study conducted by Bonaventura *et al* has shown that stem cells are primitive cells that proliferate asymmetrically, exhibit regenerative potential, and can differentiate into multiple distinct cell types. They have proven beneficial in conditions with limited therapies^[1]^. In recent years, stem cell transplantation therapy has gained attention as a promising treatment approach for neurological diseases^[6]^.

Researchers are now using neural stem cells (NSCs) for this purpose. NSCs can generate new neurons (neurogenesis) and glial cells (gliogenesis) due to their self-renewal, multipotency, and capacity for mitotic division^[1,8]^. After a neural insult, NSCs migrate and induce neurogenesis and gliogenesis, restoring the brain’s structure and function. These cells can be derived from primary tissue extraction, pluripotent stem cell differentiation, and trans-differentiation of somatic cells^[8]^.

This narrative review presents a current synthesis of the most recent experimental and clinical findings on stem cell-based therapies for major neurodegenerative disorders. In contrast to previous reviews that mainly concentrated on individual cell types or specific disease applications, this article combines evidence related to specific diseases, clinical trial results, and innovative technologies such as exosome therapy, nanotechnology, and CRISPR-based enhancements. It thus provides a holistic view that connects molecular mechanisms to practical applications. This review distinctively emphasizes the regulatory and ethical framework, tackling real-world challenges that have often been overlooked in earlier discussions.

This manuscript is made compliant with the TITAN checklist to ensure transparency in the reporting of artificial intelligence^[9]^.

## Methodology

Our team conducted a thorough search on the topic “Therapeutic Potential of Stem Cells in Neurodegenerative Diseases: Advances, Insights, and Challenges.” We searched PubMed for relevant publications from the past 5 years using the keywords “Stem cell therapy,” “Neurodegenerative diseases,” “Alzheimer’s disease,” “Parkinson’s disease,” and “Stem cell transplantation.” Meta-analyses, systematic reviews, review studies, clinical trials, and randomized controlled trials were all included. Additionally, recent clinical trials published within a year were retrieved from the Cochrane Library and ClinicalTrials.org. From the Cochrane Library, we retrieved one trial; from ClinicalTrials.org, we retrieved 11 trials; and from PubMed, we retrieved 223 studies, which were then screened for relevance. After screening, 31 studies were included in the final manuscript. Only English-language publications were included. To ensure the quality of our selection, all included papers met PICO criteria.

- Population (P): patients with NDs, including PD, AD, ALS, and stroke
- Intervention (I): stem cell therapy [mesenchymal stem cells (MSCs), adipose-derived stem cells, bone marrow stem cells, embryonic stem cells (ESCs), skeletal muscle stem cells, pluripotent stem cells, and human fetal ventral mesencephalon stem cells]
- Control (C): no treatment or standard treatment.
- Outcome (O): stem cell therapy slows disease progression, improves the patient’s quality of life, prolongs survival rate, and provides sustained clinical benefits in a wide range of NDs.

## Discussion

### Classification and characteristics of stem cells

Stem cell therapy is a treatment modality recently discovered by researchers, also known as regenerative therapy, which enhances the body’s ability to repair structurally impaired cells found in NDs and facilitates the human body’s optimal replenishment of functioning cells. The main objective of this modality is to compensate for cells targeted and lost in neurological degenerative disorders or provide environmental conditions that encourage healing. Stem cells are capable of self-regeneration and multiplication and enter different cell differentiation pathways^[10]^. Stem cells utilized for this purpose can be categorized into the following types: ESCs, induced pluripotent stem cells (iPSCs), MSCs, and NSCs^[11]^.

**HIGHLIGHTS**

- Stem cell therapy offers regenerative potential for neurodegenerative diseases.
- Mesenchymal stem cells (MSCs), induced pluripotent stem cells, and neural stem cells show promise through neuroprotection and cell replacement.
- MSC-derived exosomes cross the blood–brain barrier and enhance neuron survival.
- Ethical, safety, and regulatory challenges limit current clinical translation.
- Future focus: standardized protocols, nanotech, and gene-editing optimization.

**Figure 1.**
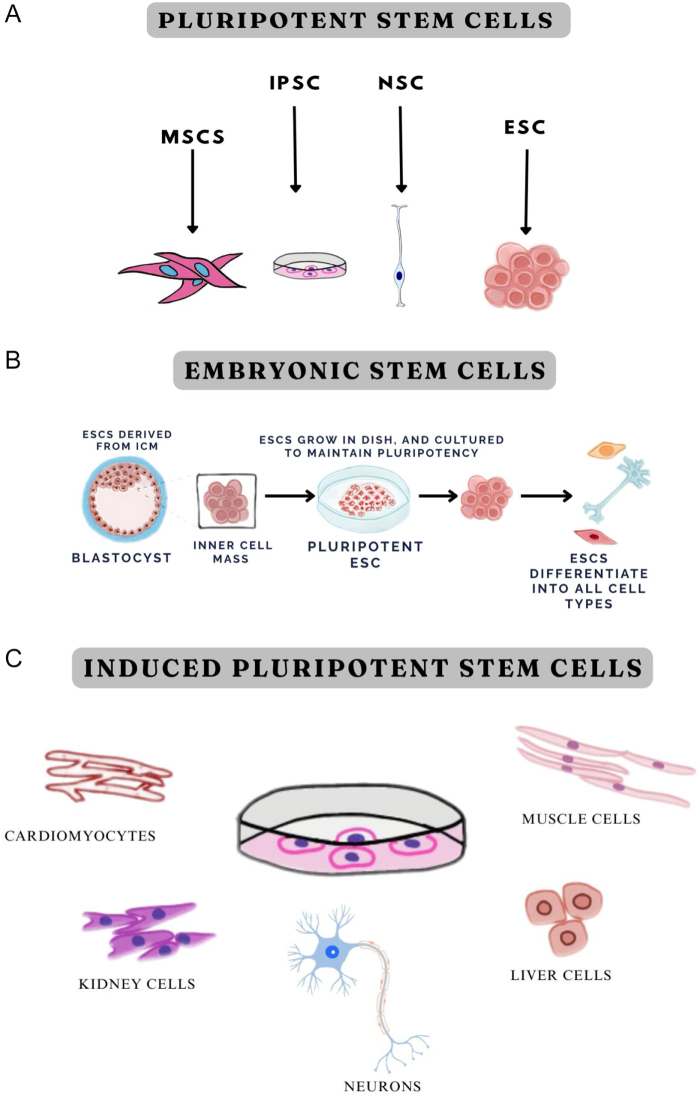
Classification and derivation of pluripotent stem cells. (A) Sources of pluripotent stem cells. (B) Process of embryonic stem cell derivation and differentiation. (C) Sources of induced pluripotent stem cells.

Figure 1(A) illustrates the sources of pluripotent stem cells.

#### ESCs

ESCs are pluripotent cells procured from embryos in their pre-implantation stage^[12]^. According to recent research, ESCs are among the most favorable stem cell types^[13]^. Due to their capacity to differentiate into nerve cells with dopaminergic characteristics, ESCs have demonstrated promising results when applied in mouse models for treating PD^[10]^. In a study on PD animal models, grafting of ESC-derived dopamine-releasing nerve cells, present at the middle stages of their differentiation, proved to be most beneficial^[14]^. Despite their desirable effects, ESCs exhibit a strong interrelation with tumorigenesis and teratoma formation, as these cells can proliferate indefinitely^[15]^. In addition to this drawback, these stem cells are also associated with the rejection of implanted stem cells, and controversies regarding the acceptability of retrieving these cells from human embryos arise, offering further challenges in this therapeutic modality^[16]^. Figure 1(B) illustrates the derivation of ESCs from the blastocyst’s inner cell mass and their differentiation into various cell types, highlighting their role in regenerative medicine.

#### iPSCs

Another class of such cells is iPSCs, which are secured by artificial methods such as the manipulation of genetic material from cells that do not possess the ability to differentiate along multiple lineages, including fibroblasts, liver parenchymal cells (hepatocytes), keratinocytes, and circulating immune T cells. These cells can mature into neuronal cells, which play a potential role in treating degenerative neurological morbidities^[10]^. Obtained from the patient’s somatic cells, stem cell therapy involving iPSCs raises no ethical debates, and it also reduces the risk of rejection after grafting, as it carries the patient’s own genome. It is a profitable resource when used in regenerative medicine, disease simulation, and drug screening. iPSCs can infinitely mature into two-dimensional (2D) and three-dimensional (3D) nerve cells, nearly identical to those found in the human brain^[16]^. A study conducted by Sivandzade *et al* has demonstrated that iPSCs transplanted into PD model systems integrated exceptionally well with the host’s system, and various studies have shown that when programmed to differentiate along a neural lineage, particularly into dopamine-releasing neurons, iPSCs alleviated functional impairments and facilitated cell intermingling in vivo. However, the maturation of iPSCs into nerve cells is found to be more complex than that of ESCs^[10]^. Figure 1(C) illustrates the sources of iPSCs.

#### MSCs

**Figure 2.**
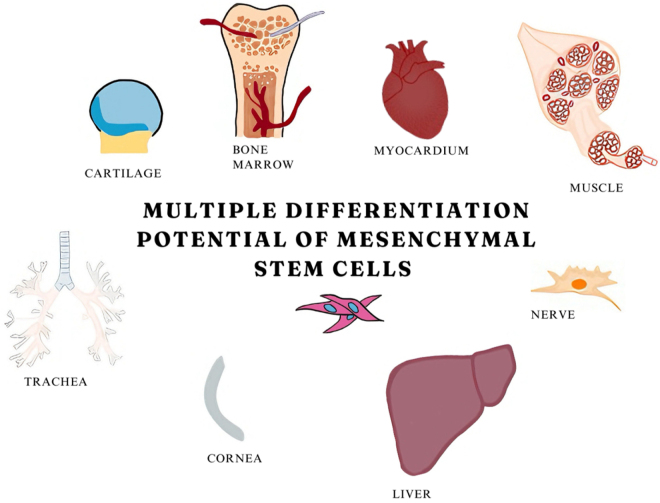
Differentiation potential of mesenchymal stem cells.

MSCs can be acquired from various tissue sources, including bone marrow, adipose tissue, and Wharton’s jelly, which is found in the umbilical cord of neonates. Figure 2 shows the differentiation potential of MSCs. MSCs are ideal for therapeutic applications, offering advantages such as their ability to differentiate into mature cells of various specialized tissues, high graft viability, ease of retrieval, and minimal controversy. The release of factors that enhance neural health may activate host stem cells to differentiate and provide protection to restored nerve cells against cell death (apoptosis) induced by stress^[17]^. Reassuring results were achieved after conducting a study with mouse models of PD as subjects, as they showed mitigation of symptoms^[18]^. MSCs used in a study demonstrated an augmentation in autophagy, providing a protective effect in an AD mouse model by increasing the removal of amyloid-β plaques, the primary pathogenic components of AD^[19]^.

#### NSCs

**Figure 3.**
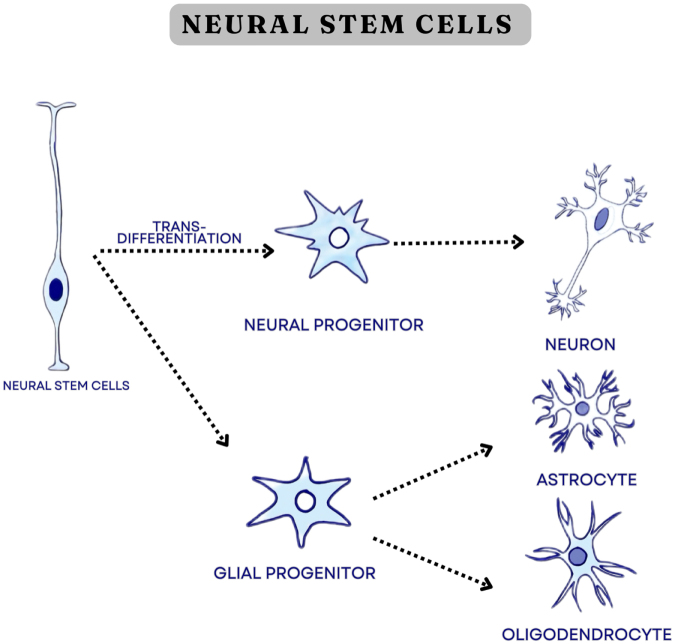
Differentiation pathway of neural cells into neurons, astrocytes, and oligodendrocytes.

NSCs can be directly extracted from the brain, as a limited number of them persist in the subventricular zone from youth, or can be obtained under specific conditions from pluripotent stem cells^[14]^. NSCs sourced from human brain cells have become an alternative treatment modality option for AD. The implantation of these cells promotes the release of a nerve growth factor, which stimulates maturation into multiple neuronal cells. This enhances neuronal cell life, regenerates learning capability, and improves the ability of neurons to modify their connections^[16]^. NSCs also contribute to the production of glial cells by secretion of biologically active substances that regulate nerve cell stimulation, synaptic function, and adaptation^[10]^. However, the process of transplanting NSCs can be challenging, as these cells must be acquired from fetal brains, thus needing manipulation of human fetuses, raising ethical concerns^[20]^. Figure 3 illustrates the differentiation pathway of neural cells.

### Delivery methods and transplantation

Administration routes for stem cells include the intravenous route, which has been proven to be comparatively beneficial; the intrahippocampal and intracerebroventricular methods, which are not convenient as they do not allow for multiple doses of transplantation; and the intranasal route, which is non-invasive and can be performed repeatedly^[21]^.

### Mechanisms of action

The alternatives used in regenerative medicine restore health in patients affected by NDs through various modes of action. MSCs contribute to this effect by releasing factors that demonstrate neurotropism, including brain-derived neurotrophic factors, glial-cell-derived neurotrophic factors, and nerve growth factors. These inhibit the apoptotic cell death of nerve cells and foster neurogenesis by secreting chemicals that induce mitosis and angiogenesis^[18]^. NSCs are responsible for producing nerve cells and supporting nervous tissue cells (glial cells), as well as self-renewal and self-restoration, during the development of the central nervous system^[14]^. IPSCs also exhibit similar effects, contributing to neurogenesis and compensating for neurons affected by neurodegenerative disorders^[10]^. ESCs possess a limitless potential to differentiate along several lineages^[22]^. iPSCs rarely exhibit tissue rejection patterns because they are derived from the host’s own cells and are easier to acquire from sources than ESCs due to ethical disputes^[10]^. However, iPSCs significantly increase the risk of developing teratomas, which is an unexpected finding when other stem cell types are experimented with^[19]^. Table 1 summarizes the characteristics of various stem cell therapies and their corresponding clinical trial stages^[23]^.

**Table 1:** Comparative summary of different stem cell types and their clinical trial stages

Stem cell type	Source	Differentiation potential	Ethical concerns	Tumor risk	Current clinical trial stage
Embryonic stem cells	Inner cell mass of the blastocyst	Pluripotent	High	High	Phase 1
Induced pluripotent stem cells (iPSCs)	Adult somatic cells	Pluripotent	Low	High	Phase 1/2
Mesenchymal stem cells	Wharton’s jelly (umbilical cord), bone marrow, adipose tissue	Multipotent	Low	Low–moderate	Phase 2
Neural stem cells	Derived from PSCs or fetal brain tissue	Multipotent	Moderate	Low–moderate	Phase 1/2
Hematopoietic stem cells	Bone marrow, cord blood	Multipotent	Low	Low	Phase 1/2

### Disease-specific evidence

#### PD

MSCs and exosomes derived from these cells have shown positive results in PD^[24]^. MSC-derived exosomes are capable of traversing the selectively permeable blood–brain barrier (BBB) and contain various proteins, including neuroplastin, vinculin, and drebrin. This method elevates dopamine levels, inhibits dopaminergic neuron death, stimulates nerve cell and myelin sheath production, and protects neural components, as observed in experimental rats^[25]^. A clinical trial for dopaminergic neurons derived from allogeneic iPSC commenced in 2018^[26]^. The Kyoto trial, conducted by Takahashi in 2020 and completed in 2023, used allogeneic iPSCs to derive dopamine (DA) progenitors, which were then purified by CORIN antibody-based cell sorting. The results showed no tumor growth nor graft induced dyskinesia, and three patients even showed motor improvement, making stem cell therapy with PSCs in PD promising, although much cannot be said about its efficacy^[27]^.

#### AD

A study was conducted, and the results concluded that MSCs exhibited increased autophagocytic activity, aiding in the effacement of amyloid-β plaques, which play a pivotal role in the pathogenesis of AD. These cells also appear to supplement the activation of supporting nerve tissue (microglia)^[19]^. iPSCs have also shown encouraging results in AD by maturing into cholinergic neurons after a study was conducted with mouse models as subjects. This outcome can also be witnessed when working with NSCs^[10]^. A clinical trial involving MSC-EVs via nasal administration had shown improvement in cognitive function by evaluation using the Montreal Cognitive Assessment and Mini-Mental State Examination, which suggests the use of stem cell EVs was successful in AD patients^[28]^. Supplemental Digital Content Figure S1, available at: http://links.lww.com/MS9/B48, illustrates Alzheimer’s pathology, characterized by the presence of amyloid plaques and neuronal loss.

#### Huntington’s disease

A rare disease resulting from an autosomal dominant mutation in the huntingtin (HTT) protein, due to trinucleotide repeats of CAG, which causes loss of GABAergic neurons in the striatum. Currently, there is no known cure or effective treatment available, which is why stem cell therapy is drawing interest^[29]^.

The preferred choice of stem cells in the treatment of Huntington’s disease (HD) is NSCs^[10]^. NSCs can be derived from the patient’s somatic cells, the brain, or even ESCs, with HD animal models providing significant evidence in support, although preclinical and clinical trials are still in the early stages. Although we can produce iNSCs from somatic cells of HD patients, the CAG repeats persist, which can be replaced by gene editing technologies such as CRISPR/Cas9^[29]^. MSCs also play a role in alleviating disease in HD patients by enhancing mitochondrial activity, ensuring cell survival, and nerve cell production^[10]^.

#### ALS

In ALS patients, specific biomarkers are identified in CSF biochemistry that decrease upon observation once the subjects are exposed to stem cell therapy^[30]^. A more challenging hurdle is encountered when stem cell therapy is employed in ALS, due to the measurable extent of motor axons and confirmation, suggesting that neurons and glial cells may influence the pathogenesis of ALS^[31]^. ESCs that differentiate into motor neurons have proven to be a powerful tool as they have shown improvement in the motor functions of ALS patients^[15]^.

NSCs derived from the spinal cords of rats and the human forebrain can be used to create motor neurons *in vitro*; these, along with neuroblasts, can form functional synapses with muscle fibers. Although ALS currently has no cure, regenerative therapy offers hope^[32]^.

### Safety and ethical concerns of stem cell therapy for NDs

#### Risks of tumor formation

Despite the promising potential of stem cell therapies, several safety and ethical concerns have become apparent with advancements in research, animal trials, and clinical trials. Stem cells can differentiate and divide indefinitely, raising concerns about their potential to form tumors. iPSCs are tumorigenic due to several factors, including incomplete differentiation, active reprogramming, and genetic mutations^[16]^. Teratomas, tumors composed of multiple tissue types, have been implicated in stem cell treatments due to their ability to differentiate into various cell types^[14]^. MSCs are considered low risk compared to other types because of their low tumorigenic potential; however, long-term cultures or genetically modified MSCs (such as MSC-derived exosomes) may increase this risk^[33]^. A case was reported in which a glioneuronal tumor developed after fetal NSC transplantation for treating ataxia telangiectasia, a rare neurodegenerative disorder. Although it is benign, its presence remains a serious concern. Immune rejection may also play a role in the regression of donor-derived tumors^[12]^. Immune rejection may play a role in the regression of donor-derived tumors^[12]^. However, its associated complications are highly undesirable, leading to therapeutic failure and triggering immune reactions. Exogenously administered MSCs, along with the previously mentioned adverse effects, may elicit embolic phenomena and graft-versus-host disease^[34]^. MSCs have varying immunogenic profiles depending on their source. Despite being considered low in immunogenicity, they may still trigger an immune reaction^[33]^. Cryopreserved MSCs exhibit an impaired immune response after thawing compared with freshly retrieved cells, leading to quicker destruction when exposed to blood^[35]^. In cases of allogeneic transplants, patients require close monitoring and follow-up. Certain factors should be considered before treating patients with MSCs, as they may influence immune responses, including donor age, genetic characteristics, and medical history. Recent advances, such as MSC-derived exosomes (MSC-Exos), have been introduced to retain the beneficial properties of MSCs while alleviating risks^[33]^. Immune-modulation strategies have also been tested to improve graft survival.

#### Ethical debates in hESC stem cell-based therapy

Ethical concerns have also been raised, which limit the progression of stem cell therapy. A challenging aspect of regenerative medicine is the source of extraction, with many methods involving the use of embryos, which raises debates about the moral implications of their use. Human umbilical cord-derived MSCs have been used in mouse models of AD, showing positive results by reducing glial activity markers, apoptosis, and oxidative stress. However, the use of fetal tissues raises religious and moral concerns^[18]^. Alternative methods are being explored, such as single-blastomere-derived ESCs, which show great potential as an alternative to embryo blastocysts, which have been ethically disapproved of^[12]^. Despite being a significant source for AD research, commercial cord blood banks for MSCs raise ethical concerns^[36]^.

#### High costs and accessibility issues

Another significant issue is the cost of stem cell therapy, from production to implementation. The manufacturing process must adhere to Good Manufacturing Practice standards, which necessitate the use of specialized materials, equipment, and facilities, all of which increase expenses. A study in 2020 revealed that the production costs for pluripotent stem cells alone can reach $500 000. Along with production, conducting preclinical and clinical trials requires substantial financial investments. Owing to the high costs, patients requiring stem cell therapy will have limited accessibility, with treatment costs estimated to range between $30 000 and $100 000 per patient^[1]^. As technological developments improve and the process becomes more efficient, costs may decrease, increasing accessibility to stem cell therapy.

### Challenges, limitations, and future directions in stem cell therapy

The Clustered Regularly Interspaced Short Palindromic Repeats (CRISPR)-Cas system is a gene-editing tool that utilizes an RNA sequence to target specific DNA sequences. Once this is done, this system works either by integrating an externally derived nucleic acid fragment into the patient’s genome or scissoring out a mutated gene sequence. This technology can also be used to create disease models. For example, an *in vitro* model of dilated cardiomyopathy was formed by introducing the RBM20 mutation into iPSCs derived from humans. CRISPR-Cas was used to fit this mutation during the pluripotent stage, and these cells later differentiated into cardiomyocytes. This lays the groundwork for gene-editing techniques used to engineer stem cells tailored to each patient’s genetic material, thereby providing a more precise and definitive response in all individuals. Moreover, this system also plays a role in controlling the expression of therapeutic factors in the host, which may bring about alleviating effects^[37]^. Another discovery that has proven beneficial in NDs is the manipulation of biomaterials in combination with cell implantation, providing more favorable conditions for healing. This way, regenerative medicine contributes more to restoring neuronal function because cells have a higher chance of surviving, rebuilding, and contributing to the reformation of neuronal circuits^[38]^. Materials used for this purpose include hydrogels, microspheres, nanoliposomes, and scaffold micro needles^[39]^. When combined with stem cell therapy as a single mode of treatment, these apparatuses can enhance therapeutic outcomes^[38]^. Stem cell therapy has yet to reach its full potential, necessitating the development of new strategies to further enhance its progress. Safety concerns must be minimized to maximize the therapeutic efficacy. Optimizing stem cell therapy requires determination of the correct dose, timing, and delivery method. As a person ages, there is a decrease in their stem cell function, resulting in lower efficacy of stem cell therapy; a significant concern is the development of abnormal DNA methylation patterns and histones, which prevent PSCs from differentiating into the desired cells. Factors such as mechanical stress, loss of extracellular matrix, and deprivation of nutrients lead to remarkable cell death. Additionally, the ability to combine stem cells into a functional neural network is a hurdle which can be overcome, as studies suggest that the application of electrical signals improves synapses between existing and transplanted neurons^[26]^. A major limitation is the poor survival rate of stem cells after transplantation, which needs to be addressed to improve therapeutic outcomes. Long-term follow-up is an aspect that should be a focus for upcoming clinical trials, particularly in cases where adverse effects may become apparent later^[26]^.

#### Optimization and preconditioning strategies

Preconditioning strategies are currently being developed to enhance the function of stem cells. Stem cells derived from specific sources, such as bone marrow and adipose tissue, have shown reduced quality and survival, respectively, which impacts their therapeutic potential and highlights the need for preconditioning strategies^[12]^. Preconditioning MSCs with an EP4 antagonist and utilizing three-dimensional culture techniques are beneficial in animal models, as they reduce inflammation and amyloid-beta accumulation, respectively^[33]^. NSCs tend to differentiate into glial cells rather than into the intended neurons. Several protein-level treatments, including viral transfection, heat pretreatment, and antibody treatment, have been evaluated for the direct differentiation of neurons^[18]^. Tumor-related transcription factors, such as c-Myc, should be avoided during reprogramming to reduce the risk of tumor formation^[16]^. The combination of stem cell therapy with nanotechnology is a promising approach. Nanoparticles improve targeting, reduce oxidative stress, enhance regenerative abilities, and improve the safety and efficacy of stem cell therapy for NDs^[12]^.

#### Advances in stem cell delivery and differentiation

Ensuring that stem cells reach their intended targets is crucial for therapeutic action, making the method of delivery an important factor. Traditional methods, such as intranasal, direct brain transplantation, and intravenous administration, have been used, but a novel approach has emerged that targets stem cells in the gut. This is based on the idea that gut dysbiosis precedes neurodegeneration and that improving the gut microbiome may enhance cognitive function^[40]^. If proven effective, this method provides a less invasive alternative to direct brain transplantations. The use of scaffolds is another therapeutic advancement that has improved stem cell delivery and survival. Additional manufacturing techniques are gaining importance for creating precision scaffolds, which could become a critical aspect of regenerative medicine^[41]^. As previously mentioned, MSC-derived exosomes (MSC-Exos) are considered superior to MSCs; however, challenges remain in their isolation, purification, and storage due to the risk of cross-contamination with lipoproteins and viruses. Further research is needed to develop standardized and efficient purification techniques for MSC-Exos, ensuring their safety and efficacy^[33]^. MSC-Exos contain molecules that improve spatial learning, reduce memory loss, and restore hippocampal neuron function. However, further experimental studies are required before humans can use them^[25]^. The ability of stem cells to differentiate into various cell types presents challenges but also allows them to respond dynamically to their environment and address all aspects of neurodegeneration. Predifferentiated cells have been used to target specific deficits, reducing the risk of uncontrolled proliferation and making therapy safer^[12]^. Single-cell RNA sequencing (scRNA-Seq), a technology that allows researchers to obtain transcriptome data, may be employed for manufacturing and release criteria, helping researchers understand cellular functions before transplantation^[42]^. CRISPR-Cas9, a gene editing technology, holds promise for enhancing the safety and effectiveness of stem cell therapy. It has been used to correct genetic mutations in iPSCs derived from patients before transplantation, creating personalized treatments tailored to specific diseases^[1]^. However, gene editing raises ethical and safety concerns and requires careful regulation.

#### Standardization of procedures

Standardization of protocols is essential for stem cell therapy to become a reliable clinical treatment option. Inconsistent therapeutic outcomes arise owing to variability in isolation techniques, culture conditions, and transplantation methods^[21]^. As mentioned above, patients receiving stem cell therapy should be monitored long-term, as certain adverse effects may take years to manifest. Continuous monitoring is crucial for improving safety by detecting immune reactions, tumor formation, and other complications^[12]^. The variability in the blood compatibility of MSCs derived from different tissues can lead to adverse effects, such as thrombosis and embolism, underscoring the clinical need for new blood compatibility assessment criteria^[35]^. The accessibility of stem cell therapy may soon improve because of the involvement of large biotech companies. With iPSC-derived cells obtained through industrial manufacturing and the formation of biobanks, clinical studies can be conducted on a larger scale, accelerating research on stem cell therapy^[14]^. A criterion to select suitable candidates should be established based on their genetics, stage of disease, and level of damage^[26]^.

#### Recent clinical trials

Table 2, which presents the results of clinical trials, has been included in this study. It encompasses a diverse range of stem cell types being evaluated for various NDs. Some clinical trials are still ongoing, and due to the unavailability of results, the expected outcome, along with what is to be evaluated/measured, has been mentioned. The results of these clinical trials will be highly advantageous for determining safety, efficacy, and appropriate dosage for use in stem cell therapy in human patients with NDs.

**Table 2:** Overview of clinical trials investigating mesenchymal stem cell therapy in neurodegenerative diseases.

Clinical trial phase	Year of publication/study completion date	Cell type used	Disease investigated	Outcomes	NCT number
N/A	2027-07-31 (expected completion date)	Bone marrow-derived mesenchymal stem cells (MSCs)	Progressive supranuclear palsy	Assessment of neurologic function post-treatment, Neurology Quality of Life encompassing activities like communication, anxiety, depression, emotional and behavioral dyscontrol, mobility, sleep disturbance, and cognitive function	NCT02795052
Phase II	2023-07-30 (completion date)	Bone marrow-derived MSCs	Parkinson’s disease (PD)	Selection of the safest and most effective number of repeat doses of allogeneic MSC infusions	NCT04506073
Phase I	2019-09-18 (actual completion date)	Bone marrow-derived MSCs	PD	Post-treatment, no serious adverse reactions related to the infusion and no responses to donor-specific human leukocyte antigens	NCT02611167
N/A	2022-09-15 (completion date)	Platelet-rich plasma + peripheral blood-derived very small embryonic-like stem cells	PD	Evaluation of the improvement in the Unified Parkinson’s Disease Rating Scale (UPDRS), Hospital Anxiety and Depression Scale, and self-report Parkinson’s Disease Questionnaire-39	NCT06142981
Phase II	2023-02-06 (completion date)	Hope Biosciences – adipose-derived MSCs (AD-MSCs)	Parkinson” Disease	Testing the efficacy and safety by changes from baseline in the Movement Disorder Society-sponsored revision of the Unified Parkinson’s Disease Rating Scale (MDS-UPDRS) Part-II score, various CBC parameters and comprehensive metabolic panel values, changes in blood pressure, weight, vital signs and coagulation panel values.	NCT04928287
Phase I/II and IIa	2013-03 (completion date) 2015-09 (actual completion date)	MSCs were isolated from the patients’ bone marrow, expanded *ex vivo*, and induced to differentiate into MSC- neurotrophic factor (NTF) cells.	Amyotrophic lateral sclerosis (ALS)	The patients treated with MSC-NTF cells in the trial demonstrated some systemic immunologic response to MSC-NTF cell treatment.	NCT01051882 And NCT01777646.
Phase I	2023-04-25 (completion date)	Umbilical cord-derived, allogeneic hMSC	Mild to moderate Alzheimer’s disease	Includes treatment-emergent serious adverse effects within 1 month post-infusion. Assess cognitive function, depressive symptoms, and quality of life up to week 65 and NPI-Q up to week 52. Biomarkers were analyzed in serum and CSF up to weeks 65 and 52. Hippocampal vol. changes were assessed via MRI at weeks 6 and 52.	NCT04040348
Phase II	2021-04-30 (actual completion date)	Human immature dental pulp stem cells (hIDPSC)	Huntington’s disease (HD)	BDNF-secreting hIDPSC helps restore the endogenous BDNF expression and the expression of MSN markers (DARPP32 and D2R) in the striatum and cortex of the HD rat model. More clinical studies are needed.	NCT03252535
N/A	2030-12(Ongoing)	Human iPSC-derived pluripotent stem cells	Neurodegenerative diseases (e.g., PD and Alzheimer’s disease, diabetic neuropathy, stroke, and spinal cord injury)	Expected outcomes include improving reprogramming methods to enhance safety and efficiency and addressing challenges related to retroviral vectors and oncogenic factors. Seeks to create a platform for studying disease mechanisms and facilitating drug screening and development.	NCT00874783
Early Phase I	2023-02-08 (completion date)	Neural, mesenchymal, and hematopoietic stem cells, and neural cells derived from embryonic stem cells and iPSCs cells.	Neurodegenerative diseases (NDs)	Safety and efficacy were evaluated by measuring the number of participants experiencing adverse effects and serious adverse effects over 12 months. Changes in the UPDRS, Hoehn and Yahr scale, Parkinson’s Disease Questionnaire, and Schwab and England score. Tracking Levodopa equivalent daily dose, cranial dopamine transporter levels, glucose metabolism, and biochemical indicators	NCT04414813
Phase 1 Phase 2	2022-03-02 (actual completion date)	MSCs from adipose tissue	ALS	Evaluation of the safety of administration of 3 doses of intravenous AD-MSCs	NCT02290886
Phase I	2019-01-31 (completion date)	MSCs	ALS	Evaluation of the safety of intraspinal delivery of MSCs in a dose-escalation study	NCT01609283
Phase I	2017-04-10 (completion date)	Bone-marrow- derived MSCs	ALS	Safety studies of Human Leukocyte Antigen (HLA) – haplo-matched allogenic bone-marrow-derived stem cells	NCT01758510
Phase I	2016-01(completion date)	Adipose tissue-derived stem cells	ALS	Assessment of the efficacy of brain transplants of autologous adipose tissue-derived stem cells	NCT02383654
Phase I Phase II	2013-08 (actual completion date)	Bone marrow-derived from MSCs	ALS	A possible clinical benefit that lasted safely for at least 6 months through switching from pro-inflammatory to anti-inflammatory conditions	NCT01363401
Phase III	2020-09-29 (completion date)	NurOwn (MSC-NTF) secreting MSCs	ALS	Number of participants whose disease progression halted or improved in post- vs pretreatment slope in Amyotrophic Lateral Sclerosis Functional Rating Scale–Revised (ALSFRS-R) (used to determine participants’ assessment of their capability and independence in 12 functional activities) score in NurOwn treatment vs placebo	NCT03280056
Phase I	2026-12-31 (Estimated completion date)	MSCs preconditioned with ethionamide	Frontotemporal dementia	To see safety and potential efficacy after 4 weeks of 3rd intraventricular injection, determine dose limiting toxicity	NCT05315661
Phase II	2013-07 (completion date)	Umbilical cord MSCs	Hereditary cerebellar ataxia	Evaluation by blood tests and nerve function to see whether it is clinically safe and valid treatment for hereditary cerebellar ataxia	NCT01489267
Early Phase I	2026-04-30 (Estimated completion date)	Aleeto, a nerve repair protein derived from cellular exosomes secreted by stem cells under emergency conditions	Multiple systems atrophy	Potential for promoting endogenous neural tissue repair and exhibiting significant neuroprotective and neurorestorative effects	NCT06765733
N/A	2024-07-01 (completion date)	Stem cells from human exfoliated deciduous teeth- conditioned media (SHED-CM)	ALS	The progression rate in the ALSFRS-R score was slower, suggesting a delay in disease progression.	NCT06608719
Phase I	2028-08-31 (estimated completion date)	Human umbilical cord MSCs	Multiple NDs such as Alzheimer’s, Parkinson’s, multiple system atrophy, Lewy body dementia, and frontotemporal dementia	Evaluate efficacy and safety in the various NDs	NCT06607900
N/A	2026-09-30 (estimated completion date)	Human amniotic mesenchymal cell secretome	In ALS, Multiple Sclerosis, and healthy volunteers	Demonstration of immunomodulatory and pro-regenerative potential of hAMSC to counteract neurodegeneration	NCT06551649
Phase I	2026-12-31 (estimated completion date)	Induced neural stem cells derive DA precursor cells	PD	The safety, tolerability, evidence of cell survival, and the efficacy on PD symptoms by assessing changes during medication “off” time in the MDS-UPDRS	NCT05901818
Early Phase I	2023-08-30 (completion date)	Human amniotic epithelial stem cells	PD	The therapeutic safety and effectiveness of multiple treatments will be evaluated to develop an optimal stem cell treatment strategy.	NCT05435755
Phase II	2021-04-30 (completion date)	MSCs	HD	Identify the dose of the product that provides the best clinical response Motor assessment will be performed with the Unified Huntington’s Disease Rating Scale (UHDRS) scale, and improvement will be evaluated	NCT03252535
Phase II	2015-09 (actual study completion date)	MSCs	ALS	The results indicate that the administration of MSC-NTF cells via IT and IM methods in ALS patients is safe and may offer possible clinical benefits.	NCT01777646
Phase II	2020-12-31 (actual study completion date)	MSCs	ALS	Study findings suggest that repeated intrathecal injections of autologous MSCs were safe for ALS patients and showed potential medium-term clinical benefits, which appeared to be associated with the timing between cell administrations. Additional large-scale studies are necessary to verify these results.	NCT04821479
Phase I	2011-04 (actual completion date)	MSCs	ALS	AD-MSCs show promise in treating various conditions, prompting research on their safety, efficacy, and mechanisms. Single-cell profiling helps identify markers and supports standardized manufacturing and clinical monitoring.	NCT01142856
Phase II	2016-07 (actual completion date)	MSCs- NTF cells	ALS	The transplantation of MSC-TNF cells is safe and has shown early indications of potential benefit. This sets the stage for a future clinical trial involving multiple doses of intrathecal autologous MSC-NTF cell transplants in ALS patients.	NCT02017912
Phase I/IIa	2019-10-18 (actual study completion date)	Human neural progenitor cells transduced with GDNF	ALS	No cell dose had an effect on disease onset. Extended data and a human-specific antibody revealed a large region of GDNF.	NCT02943850
Phase I/IIa	2017 (year of publishing)	MSCs	Ataxia	All subjects demonstrated good tolerance to the procedures and successfully completed the study as planned.	NCT01649687
Phase I/II	June 2016 (year of publishing)	AD-MSCs	Ataxia	Data indicate that IV administration of Stemchymal is safe for all patients and provides short-term therapeutic benefits in those with SCA.	NCT02540655
Phase I	2024-03-31 (study completion date)	iPSCs	ALS	A study evaluates the efficacy and long-term safety of bosutinib, a Src/c-Abl inhibitor, as a treatment for ALS following its phase I safety confirmation.	NCT04744532

#### Failed/withdrawn clinical trials

Results of preclinical/animal studies have shown promising results throughout the years, making SCT a valuable and revolutionary treatment option for those suffering from NDs. However, preclinical findings can be unreliable and may not yield similar results when applied clinically^[32]^. Furthermore, conducting clinical trials is not an easy task, with issues such as stereotactic methods resulting in respiratory problems^[43]^, insufficient funding^[44]^, improper protocol, and Covid-19^[44]^ being reasons clinical trials have been withdrawn. Strict guidelines must be followed to have a trial approved and conducted.

#### Food and Drug Administration/European Medicines Agency regulations

Multiple obstacles delay the development of SCT, which limits its potential. The U.S Food and Drug Administration (FDA) and European Medicines Agency (EMA) have guidelines that must be strictly followed involving how stem cells are isolated and transplanted, requiring standardized protocols. There is also uncertainty in classifying stem cell therapy, as it is unclear whether it falls under the category of drugs or biological products. This makes gaining approval and navigating the regulatory process significantly more difficult, costly, and time-consuming^[32]^. So far, the FDA has approved HSCs that can be extracted from the bone marrow of adults. Several clinical trials have also been approved, with informed consent obtained from every participant.

#### Unregulated stem cell clinics

**Figure 4.**
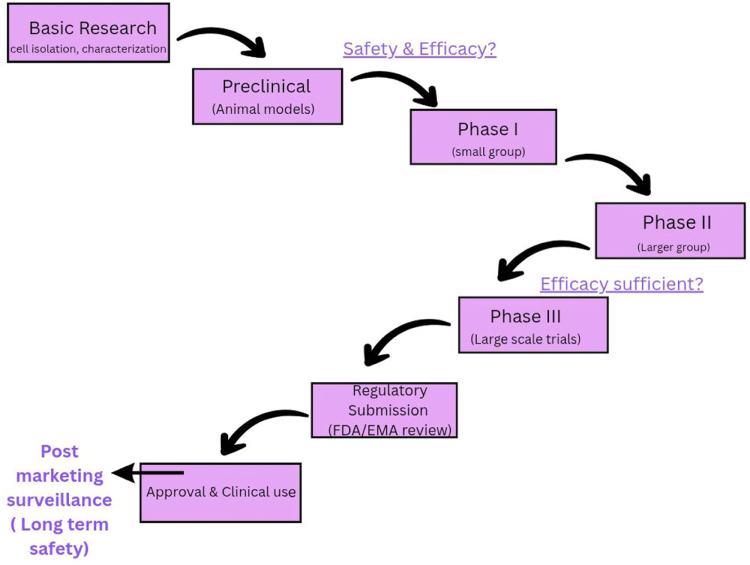
Flowchart of translational pathway from preclinical to clinical stages.

The strict guidelines regarding clinical trials and safety standards of stem cell treatments result in disapproval by the EMA and the U.S FDA, leading many facilities to provide such therapies without adequate research. Patients who have suffered long from NDs, along with spinal cord injuries and diabetes, tend to be exploited by such unregulated clinics, giving them false hope. These clinics carry unstandardized stem cell preparations without safety evaluations, resulting in complaints of tumors or infections from those who underwent treatment. Some patients may even be desperate to fly out to countries that lack control, with facilities promising access to stem cell therapy. To date, approximately 700–1000 unregulated stem cell clinics are operating worldwide, primarily in countries such as the United States, Mexico, and Thailand. International and national health organizations must collaborate to shut down these clinics, while also raising public awareness to promote safe and regulated treatment options. Legal action was taken in 2019 by the FDA against a clinic in Florida, following multiple warnings sent in 2017. Similarly, in Japan, the government imposed heavy fines on unlicensed clinics. With properly followed regulations, stem cell therapy can improve the lives of those suffering from various disorders; however, these must be globally established with strict laws in every country^[45]^. Figure 4 illustrates the translational pathway from preclinical research to clinical application^[46]^.

#### Learning points

- Therapies based on stem cells signify a transformative approach in the treatment of neurodegenerative disorders, providing regenerative advantages instead of just symptomatic relief.
- Exosomes derived from MSCs demonstrate encouraging outcomes in traversing the BBB and reducing neuroinflammation.
- Key obstacles to clinical implementation include ethical and safety considerations, particularly concerning tumor risk and immune system compatibility.
- The combination of nanotechnology, gene editing (such as CRISPR-Cas9), and biomaterials can enhance the effectiveness of treatments.
- For successful integration into standard clinical practice, it’s essential to focus on regulatory uniformity, cost reduction, and long-term patient monitoring.

## Conclusion

Neurodegenerative disorders are characterized by gradual neuronal deterioration, which frequently includes overlapping symptoms and motor deficits. Radiation, surgery, and rehabilitation are treatment methods that give symptomatic alleviation. Stem cell therapy is an emerging and promising approach for NDs. Various stem cells, including ESCs, iPSCs, MSCs, and NSCs, each offer unique benefits and challenges. Additionally, MSC-derived exosomes have shown promising results in NDs like AD and PD. However, problems such as immunological rejection, tumor risks, and ethical issues persist. Advances in delivery systems and nanotechnology are crucial to enhancing safety and efficacy, paving the way for future clinical applications. In summary, this review consolidates recent advancements and clinical trial evidence, bridging the gap between basic science and translational perspectives on stem cell therapy for NDs. By emphasizing innovative delivery methods, approaches to genetic modification, and ethical considerations, this review goes beyond the current literature to offer practical recommendations for future investigations and clinical applications.

## Data Availability

All data used in this narrative review are publicly available and sourced from previously published studies. No new data were generated for this work. All included articles have been appropriately cited within the manuscript and are available through the references section.

## Research registration unique identifying number (UIN)

Not required because it is a narrative review.

## Peer and provenance statement

Not commissioned, externally peer-reviewed.
